# Getting under your skin: The hidden dermal niche of trypanosomatid parasites

**DOI:** 10.1371/journal.ppat.1014745

**Published:** 2026-11-05

**Authors:** Kyrie P. Dickson, Annette MacLeod, Martin S. Llewellyn, on behalf of the CRUZIDERM Consortium, Walt Adamson, Julio Alonso-Padilla, Antonella Bacigalupo, Jean-Mathieu Bart, Bruno Bucheton, Jaime A. Costales, Anahí G. Díaz, Lineth Garcia, Rhiannon Heslop, Christian Hidalgo, Michael Lewis, Maria-Jesus Pinazo Delgado, Alejandra Restoy-Vasquez, Mirko Rojas, Antonieta Rojas de Arias, Brice Rotureau, Nicolás Tomasini, Etienne Waleckx, Victoria Wilde

**Affiliations:** 1 School of Biodiversity, One Health and Veterinary Medicine, University of Glasgow, Glasgow, United Kingdom; 2 School of Infection & Immunity, University of Glasgow, Glasgow, United Kingdom; 3 INTERTRYP, Université de Montpellier, Cirad, IRD, Montpellier, France; 4 ISGlobal, Barcelona, Spain; 5 CIBER de Enfermedades Infecciosas, Instituto de Salud Carlos III (CIBERINFEC, ISCIII), Madrid, Spain; 6 Fundación SANIT (Salud Naturaleza Integral), Cochabamba, Bolivia; 7 Centro de Modelamiento y Perspectiva One Health (CEMPOH), Universidad de Las Américas, Santiago, Chile; 8 Research Ring in Pest Insects and Climate Change (PIC2), Santiago, Chile; 9 Centro de Investigacion para la Salud en America Latina (CISeAL), Pontificia Universidad Catolica del Ecuador, Quito, Ecuador; 10 Instituto de Patología Experimental “Dr. Miguel Ángel Basombrío”, Facultad de Ciencias de la Salud, Universidad Nacional de Salta-CONICET, Salta, Argentina; 11 Universidad Mayor de San Simón (UMSS), Cochabamba, Bolivia; 12 Division of Biomedical Sciences, Warwick Medical School, University of Warwick, Coventry, United Kingdom; 13 Drugs for Neglected Diseases initiative (DNDi), Rio de Janeiro, Brazil; 14 Centro de Investigación Biomédica en Red de Enfermedades Infecciosas (CIBERINFEC), Instituto de Salud Carlos III, Madrid, Spain; 15 Centro para el Desarrollo de la Investigación Cientifica (CEDIC), Manduvirá 635, Asunción, Paraguay; 16 Trypanosome Transmission Group, Trypanosome Cell Biology Unit, INSERM U1347, Department of Parasites and Insect Vectors, Institut Pasteur, Université Paris Cité, Paris, France; 17 Parasitology Unit, Institut Pasteur of Guinea, Conakry, Guinea; 18 Centro de Investigaciones Regionales “Dr. Hideyo Noguchi”, Autonomous University of Yucatán, Mérida, Mexico; University of Ostrava: Ostravska univerzita, CZECH REPUBLIC

## Overview

The TriTryps—*Trypanosoma brucei*, *Leishmania* spp*.*, and *Trypanosoma cruzi*—cause three major neglected diseases: Human African Trypanosomiasis (HAT), Leishmaniasis, and Chagas disease, respectively. A central challenge in the diagnosis and treatment of these diseases is their silent, often lifelong chronic phase. While our understanding of *T. brucei* and *Leishmania* has surged in recent years, knowledge gaps in *T. cruzi* research directly hinder our ability to reliably diagnose, treat, and monitor Chagas disease—which is endemic across the Americas and spreading globally.

Most patients with Chagas disease show no symptoms for decades while the number of parasites in the blood **(parasitaemia)** drops to undetectable levels. While we know the parasite can sequester in heart and digestive tissues—leading to life-threatening complications in roughly one third of infected individuals—the full map of *T. cruzi’s* distribution in the host remains unclear, hampering diagnosis and treatment monitoring. If the parasite is not in the blood, how does its **haematophagous** (blood-feeding) insect vector continue to acquire and transmit infection? What other tissues might *T. cruzi* persist in during these silent decades?

Recent breakthroughs in *T. brucei* and *Leishmania* suggest the answer is hiding in plain sight: the skin. Here, we evaluate the skin’s role as a critical parasite reservoir and hidden transmission interface, and propose a dermal-centric roadmap for future *T. cruzi* research. Shifting the paradigm from blood to skin could guide new avenues for the improved diagnosis, treatment and control of Chagas disease.

## More than just a bite site: Lessons from *Leishmania* and *Trypanosoma brucei*

Historically, the skin was viewed as merely the entry point for trypanosomatid parasites. Once an insect vector takes a blood meal, parasites were thought to transit the skin briefly before migrating to ‘target’ tissues: the blood and central nervous system (CNS) for *T. brucei* [1]; the bone marrow, liver, and spleen for visceral *Leishmania* spp. [2]; and the heart and digestive system for *T. cruzi* [3]. While cutaneous Leishmaniasis is a clear exception, the skin was generally dismissed in systemic TriTryp infections, deemed relevant only during bite-site lesions in early-stage disease.

However, far from being a passive barrier, our skin represents the first arena of host-parasite conflict—a sophisticated, three-layered, immunological landscape (Fig 1). The outermost **epidermis** forms a protective barrier of keratinocytes and immune-sensing Langerhans cells; below, the thicker **dermis** is a collagen-rich matrix containing blood and lymph vessels, fibroblasts, and teeming with dermal dendritic cells, macrophages and lymphocytes; and the **hypodermis** provides a vast layer of subcutaneous fat where adipocytes interact with resident immune cells [4]. This environment is uniquely adapted to tolerate diverse antigens—a feature which many parasites exploit to establish and maintain infection within the skin; *Leishmania* spp. dampen protective immunity to persist inside dermal macrophages (reviewed in [2]) and *T. brucei* exploits specific skin-resident T cells and adipocytes to dampen cutaneous inflammation during chronic infection [5]. Given that the skin is a vast, immunologically distinct niche capable of harbouring parasites, could similar mechanisms underpin chronic *T. cruzi* persistence?

**Fig 1:**
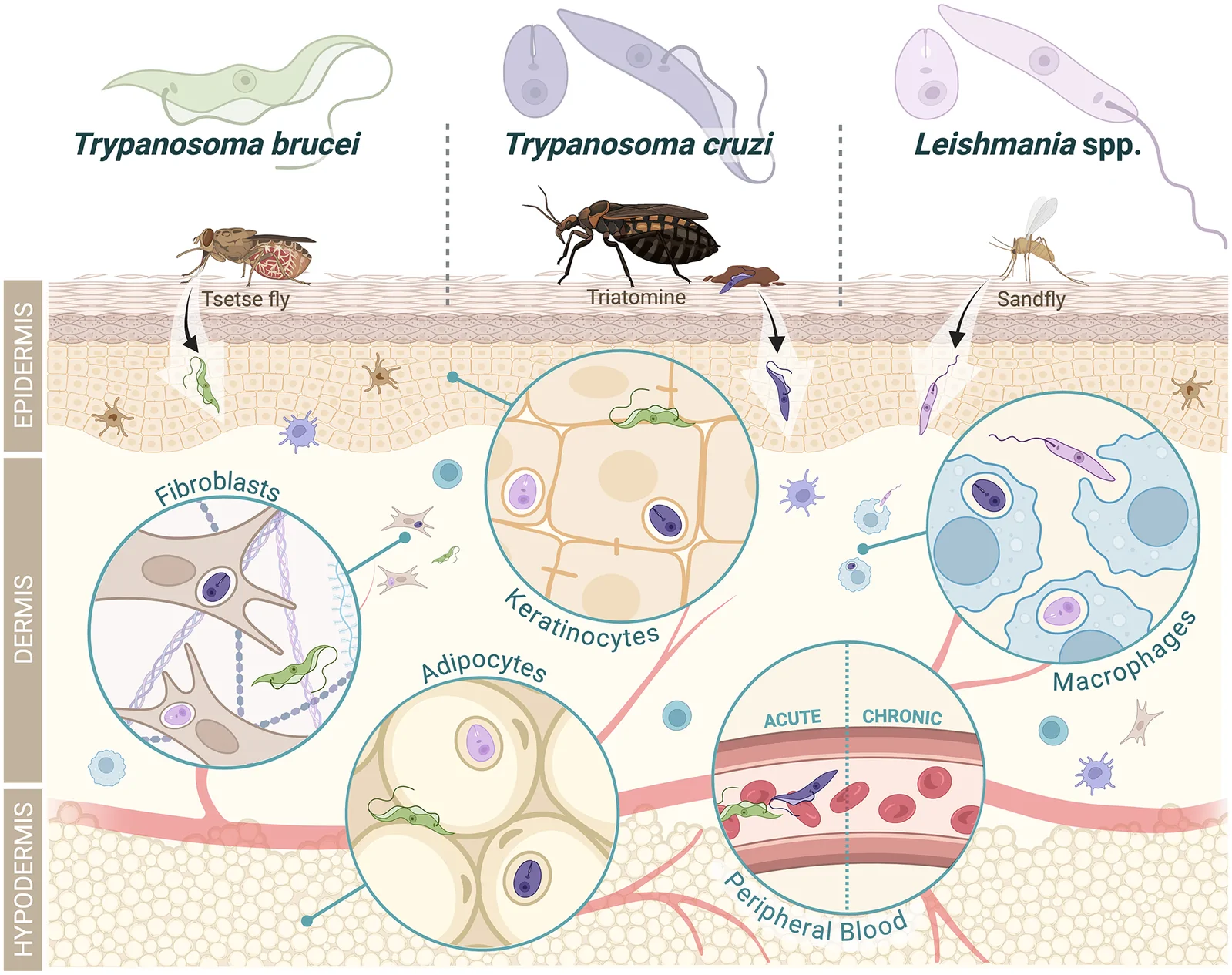
TriTryps in the skin. **(Top Panel):** Vector-mediated introduction of *Trypanosoma brucei* (tsetse fly), *Trypanosoma cruzi* (triatomine bug) and *Leishmania* spp. (sandfly) into the skin. While *T. brucei* and *Leishmania* spp. are directly inoculated through salivary transmission, vector transmission of *T. cruzi* is stercorarian, meaning the parasite is excreted in the bug’s faeces and introduced into the host through the bite wound. *T. brucei* remains an obligate extracellular parasite throughout its lifecycle, whereas both *T. cruzi* and *Leishmania* spp. have a rounded, intracellular life stage called an amastigote. **(Main panel):** Anatomical distribution of the TriTryps across the three layers of human skin: the outermost epidermis, dermis, and hypodermis. Zoom-ins highlight distinct parasite lifestyles and dermal cell tropisms: intracellular *Leishmania* and *T. cruzi* amastigotes can reside within dermal macrophages, fibroblasts, keratinocytes and adipocytes, and *T. brucei* resides extracellularly across these three skin layers. These cutaneous niches may represent critical parasite reservoirs that persist even when parasitaemia falls below detectable levels during chronic infection. *Created with BioRender* (https://BioRender.com/oc05oqn).

## *Trypanosoma brucei’s* silent skin reservoir

The most striking evidence for the skin’s role as a parasite reservoir comes from *T. brucei.* HAT was long defined by the presence of trypanosomes in the blood and CNS, until landmark studies in 2016 demonstrated that *T. brucei* colonises both the skin [6] and subcutaneous adipose tissue [7], persisting between collagen bundles and adipocytes [8]. Crucially, **xenodiagnosis**—a method where uninfected, lab-reared insect vectors are fed directly on a host’s skin—demonstrated that tsetse flies can acquire parasites directly from these skin reservoirs, even in mice with undetectable parasitaemia [6],[8]. More recent findings suggest the skin could be a key, silent reservoir facilitating transmission in humans [9], and that skin biopsies may represent a feasible HAT diagnostic strategy where blood-based methods fail [10].

This dermal population is not simply a spillover from the blood; skin-residing *T. brucei* trypanosomes represent an independent, replicative reservoir with uniquely adapted gene expression and metabolic profiles, including the upregulation of lipid metabolism genes in mouse models [7]. Skin-dwelling parasites may even employ distinct immune evasion strategies to survive in this niche. As most antigenic variation occurs not in the blood, but in the mouse tissues—including the subcutaneous adipose layer [11]—the dermis could provide an ideal niche for African trypanosomes to diversify their population while sheltered from the systemic immune response. Further, sub-populations of slow-growing parasites within visceral adipose tissue are able to survive drug treatment [12]. These findings prompt a critical question: could dermal reservoirs contribute to treatment failure in TriTryp pathology?

## Dermal persistence of *Leishmania*

Unlike the other TriTryps, some *Leishmania* spp. have a defined skin pathology known as Cutaneous Leishmaniasis (CL), where skin lesions arise from parasites replicating inside dermal macrophages [2]. However, even in Visceral Leishmaniasis (VL)—a systemic infection of the spleen, liver, and bone marrow—the skin remains a crucial, often overlooked reservoir. This is most evident in Post-Kala-Azar Dermal Leishmaniasis (PKDL), where patients ‘cured’ of systemic infection develop skin lesions laden with parasites [13].

As with *T. brucei,* xenodiagnostic studies demonstrate that skin-dwelling *Leishmania* parasites in both VL and PKDL patients are highly infectious to sandflies [14]. Strikingly, sandflies fed directly on the skin of PKDL patients—rather than indirectly from the blood—were significantly more likely to become infected, suggesting the skin acts as a potent reservoir for vector transmission [14]. Moreover, murine model xenodiagnosis and modelling directly links skin parasite burden to host infectiousness [15]. High rates of positive xenodiagnosis reflect the density of skin-resident parasites; despite containing 10 times less blood than traditional blood samples, skin biopsies yielded higher rates of *Leishmania* DNA, suggesting the skin could be a more reliable diagnostic source of parasites than the blood [16]. These findings challenge the assumption that only symptomatic, systemic cases drive outbreaks. Instead, the skin is positioned as a hidden reservoir that maintains the transmission cycle even after clinical ‘cure’.

## *Trypanosoma cruzi*: Hiding in plain sight?

This paradigm shift in HAT and Leishmaniasis research forces us to reconsider blind spots in Chagas disease research. If both *T. brucei* and visceral *Leishmania* spp. maintain a silent reservoir in the skin, could *T. cruzi* be doing the same?

Perhaps the parasite’s affinity for fat facilitates a dermal reservoir; *T. cruzi* is known to sequester in the fat surrounding the heart and colon—so why not also in the hypodermis, the vast layer of fat underlying the skin? Indeed, *T. cruzi* persists within the subcutaneous adipose tissue of both humans [17] and mice [18], whilst bioluminescent imaging demonstrates that parasites persist in the dermal tissues of over 80% of chronically infected mice and hamsters [19],[20].

Further, *T. cruzi’s* ability to infect human keratinocytes [21] and fibroblasts [22] suggests the parasite is not simply passing through the skin, but has the potential to actively integrate into the dermal environment. Clinical observations support this: in immunocompromised patients, Chagas reactivation often manifests as “reactivation chagomas”—parasite-laden skin nodules (reviewed in [23]). The sudden emergence of these lesions could suggest that parasites were not recruited from the blood, but were rather ‘lying in wait’ within local dermal or subcutaneous tissues—supported by the higher sensitivity of xenodiagnosis than traditional PCR [24]. Moreover, host cells infected with *T. cruzi* exhibit an upregulation of lipid synthesis pathways, suggesting that *T. cruzi* may be able to manipulate the host dermal environment to favour its own integration and survival [18],[25].

A dermal niche may resolve a long-standing transmission paradox in Chagas epidemiology: how do triatomines become infected when parasitaemia is almost non-existent? Perhaps the vector can ingest skin-dwelling parasites during the probing phase of feeding, before even reaching a host’s capillary. A dermal reservoir could therefore provide a transmission advantage, facilitating vector uptake of amastigotes or locally emerging trypomastigotes simply through proximity—and bypassing the need for high circulating concentrations of parasites that could otherwise trigger an attenuating host immune response. The skin could represent the essential bridge to maintain transmission of *T. cruzi* when the peripheral blood is void of parasites.

## A roadmap for the dermal frontier

Emerging clinical and experimental evidence—and a growing list of unanswered questions—highlights the urgent need to formally integrate the skin into the Chagas disease paradigm. To address these knowledge gaps, we propose a conceptual roadmap to guide future skin-centric research (Fig 2).

**Fig 2:**
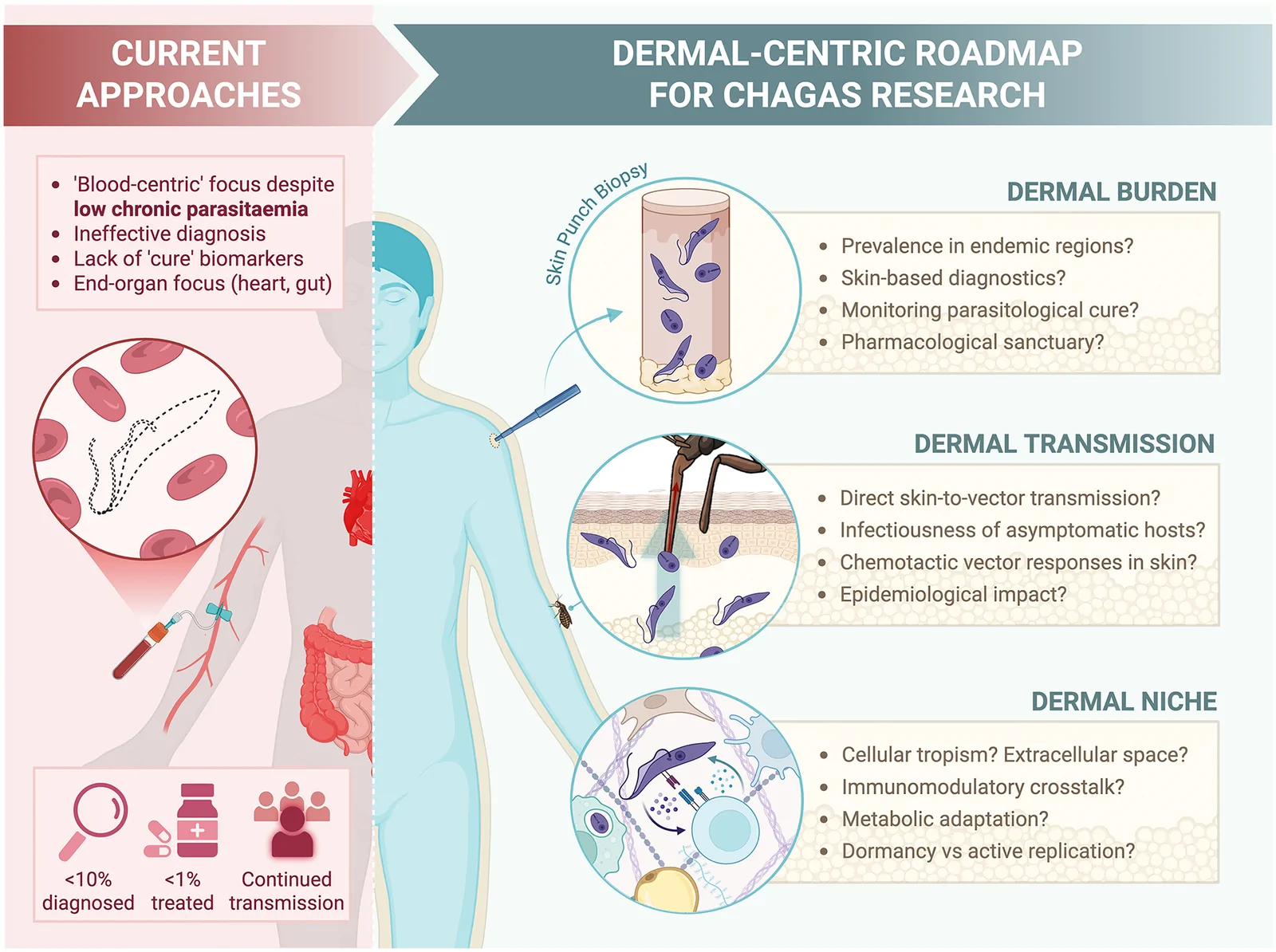
A dermal-centric roadmap for Chagas disease research. **(Left)** The Blood-Centric Paradigm: Current approaches in Chagas disease diagnosis, treatment and control rely heavily on monitoring blood circulation, despite the clinical reality that chronically infected individuals typically exhibit low or undetectable parasitaemia. This blood-centric focus leaves critical blind spots regarding parasite persistence and treatment failure, contributing to a stark public health gap where less than 10% of Chagas disease cases are diagnosed and less than 1% receive treatment. **(Right)** The Dermal Frontier: A proposed roadmap to bridge these gaps by focusing on three main investigative pillars to explore the role of the skin in Chagas disease pathology, persistence and transmission. Transitioning toward this skin-inclusive model is essential for developing effective biomarkers, refining epidemiological models, and ultimately improving Chagas disease control strategies. *Created with BioRender* (https://BioRender.com/oc05oqn).

First, the prevalence and topography of skin-dwelling *T. cruzi* parasites must be mapped in endemic populations. If the dermis acts as a reliable, long-term reservoir, validating dermal-targeted diagnostic and biomarker platforms could revolutionise how we detect active infections and monitor cure—challenges that current blood-based serology and PCR cannot fully resolve. Further, investigating whether the hypodermis acts as a pharmacological sanctuary—with restricted drug penetration or altered parasite metabolic states—is vital to understanding chronic treatment failure. Addressing this requires integrating skin pharmacokinetics into drug discovery pipelines and optimising compounds for dermal delivery.

Second, the contribution of skin-resident parasites to the triatomine transmission cycle requires urgent clarification. We must determine whether vectors can efficiently acquire infection from hosts with low systemic parasitaemia but high dermal burdens. If skin-to-vector transmission is efficient, current epidemiological models significantly underestimate the infectiousness of asymptomatic hosts, necessitating a revision of control strategies.

Finally, the cellular tropism, spatial distribution, lifecycle stages and metabolic states of *T. cruzi* within the complex landscape of the skin remain largely unknown. Characterising the immunomodulatory crosstalk between the parasite and host dermal architecture may shed light on the mechanisms of long-term persistence and tissue reactivation.

## Conclusion

Compelling evidence positions the skin as a unifying dimension of TriTryp infection dynamics. However, this niche is critically overlooked in *T. cruzi* research. Reconceptualising the skin—not merely as a transient entry point, but as a persistent parasite reservoir—could transform how we diagnose, treat and ultimately control Chagas disease.

## References

[1] Brun R, Blum J, Chappuis F, Burri C. Human African trypanosomiasis. Lancet. 2010;375(9709):148–59. doi: 10.1016/S0140-6736(09)60829-1 19833383

[2] Gupta AK, Das S, Kamran M, Ejazi SA, Ali N. The pathogenicity and virulence of Leishmania: Interplay of virulence factors with host defenses. Virulence. 2022;13(1):903–35. doi: 10.1080/21505594.2022.2074130 35531875 PMC9154802

[3] Frade AF, Guérin H, Nunes JPS, Silva LFSE, Roda VMDP, Madeira RP. Cardiac and digestive forms of Chagas disease: An update on pathogenesis, genetics, and therapeutic targets. Mediators Inflamm. 2025;2025(1):8862004. doi: 10.1155/mi/8862004 PMC12037249 40297326

[4] Kabashima K, Honda T, Ginhoux F, Egawa G. The immunological anatomy of the skin. Nat Rev Immunol. 2019;19(1):19–30. doi: 10.1038/s41577-018-0084-5 30429578

[5] Quintana JF, Sinton MC, Chandrasegaran P, Lestari AN, Heslop R, Cheaib B, et al. γδ T cells control murine skin inflammation and subcutaneous adipose wasting during chronic Trypanosoma brucei infection. Nat Commun. 2023;14(1):5279. doi: 10.1038/s41467-023-40962-y 37644007 PMC10465518

[6] Capewell P, Cren-Travaillé C, Marchesi F, Johnston P, Clucas C, Benson RA. The skin is a significant but overlooked anatomical reservoir for vector-borne African trypanosomes. eLife. 2016;5:e17716. doi: 10.7554/eLife.17716 PMC5065312 27653219

[7] Trindade S, Rijo-Ferreira F, Carvalho T, Pinto-Neves D, Guegan F, Aresta-Branco F, et al. *Trypanosoma brucei* parasites occupy and functionally adapt to the adipose tissue in mice. Cell Host Microbe. 2016;19(6):837–48. doi: 10.1016/j.chom.2016.05.002 27237364 PMC4906371

[8] Caljon G, Van Reet N, De Trez C, Vermeersch M, Pérez-Morga D, Van Den Abbeele J. The dermis as a delivery site of *Trypanosoma brucei* for tsetse flies. PLoS Pathog. 2016;12(7):e1005744. doi: 10.1371/journal.ppat.1005744 27441553 PMC4956260

[9] Capewell P, Atkins K, Weir W, Jamonneau V, Camara M, Clucas C, et al. Resolving the apparent transmission paradox of African sleeping sickness. PLoS Biol. 2019;17(1):e3000105. doi: 10.1371/journal.pbio.3000105 30633739 PMC6345479

[10] Camara M, Soumah AM, Ilboudo H, Travaillé C, Clucas C, Cooper A. Extravascular dermal trypanosomes in suspected and confirmed cases of gambiense human African trypanosomiasis. Clin Infect Dis. 2021;73(1):12–20. doi: 10.1093/cid/ciaa897 PMC8246823 32638003

[11] Beaver AK, Keneskhanova Z, Cosentino RO, Weiss BL, Awuoche EO, Smallenberger GM, et al. Tissue spaces are reservoirs of antigenic diversity for *Trypanosoma brucei*. Nature. 2024;636(8042):430–7. doi: 10.1038/s41586-024-08151-z 39478231 PMC11634766

[12] Trindade S, De Niz M, Costa-Sequeira M, Bizarra-Rebelo T, Bento F, Dejung M, et al. Slow growing behavior in African trypanosomes during adipose tissue colonization. Nat Commun. 2022;13(1):7548. doi: 10.1038/s41467-022-34622-w 36481558 PMC9732351

[13] Zijlstra EE, Musa AM, Khalil EAG, el-Hassan IM, el-Hassan AM. Post-kala-azar dermal leishmaniasis. Lancet Infect Dis. 2003;3(2):87–98. doi: 10.1016/s1473-3099(03)00517-6 12560194

[14] Mondal D, Bern C, Ghosh D, Rashid M, Molina R, Chowdhury R, et al. Quantifying the infectiousness of post-kala-azar dermal leishmaniasis toward sand flies. Clin Infect Dis. 2019;69(2):251–8. doi: 10.1093/cid/ciy891 PMC6603265 30357373

[15] Doehl JSP, Bright Z, Dey S, Davies H, Magson J, Brown N, et al. Skin parasite landscape determines host infectiousness in visceral leishmaniasis. Nat Commun. 2017;8(1):57. doi: 10.1038/s41467-017-00103-8 28680146 PMC5498584

[16] Kirstein OD, Abbasi I, Horwitz BZ, Skrip L, Hailu A, Jaffe C, et al. Minimally invasive microbiopsies: A novel sampling method for identifying asymptomatic, potentially infectious carriers of *Leishmania donovani*. Int J Parasitol. 2017;47(10–11):609–16. doi: 10.1016/j.ijpara.2017.02.005 28455239 PMC5596977

[17] Ferreira AVM, Segatto M, Menezes Z, Macedo AM, Gelape C, de Oliveira Andrade L, et al. Evidence for *Trypanosoma cruzi* in adipose tissue in human chronic Chagas disease. Microbes Infect. 2011;13(12–13):1002–5. doi: 10.1016/j.micinf.2011.06.002 21726660 PMC3552247

[18] Combs TP, Nagajyothi, Mukherjee S, de Almeida CJG, Jelicks LA, Schubert W, et al. The adipocyte as an important target cell for *Trypanosoma cruzi* infection. J Biol Chem. 2005;280(25):24085–94. doi: 10.1074/jbc.M412802200 15843370

[19] Lewis MD, Francisco AF, Jayawardhana S, Langston H, Taylor MC, Kelly JM. Imaging the development of chronic Chagas disease after oral transmission. Sci Rep. 2018;8(1):11292. doi: 10.1038/s41598-018-29564-7 PMC6062536 30050153

[20] Langston H, Fortes Francisco A, Doidge C, Roberts CH, Khan AA, Jayawardhana S. Dynamics of *Trypanosoma cruzi* infection in hamsters and novel association with progressive motor dysfunction. PLoS Negl Trop Dis. 2024;18(6):e0012278. doi: 10.1371/journal.pntd.0012278 PMC11221660 38905323

[21] Kroh K, Barton J, Fehling H, Lotter H, Volkmer B, Greinert R, et al. Antimicrobial activity of NK cells to *Trypanosoma cruzi* infected human primary Keratinocytes. PLoS Negl Trop Dis. 2024;18(7):e0012255. doi: 10.1371/journal.pntd.0012255 PMC11262665 39038032

[22] Oliveira AER, Pereira MCA, Belew AT, Ferreira LRP, Pereira LMN, Neves EGA, et al. Gene expression network analyses during infection with virulent and avirulent *Trypanosoma cruzi* strains unveil a role for fibroblasts in neutrophil recruitment and activation. Hill KL, editor. PLOS Pathog. 2020 Aug 18;16(8):e1008781. doi: 10.1371/journal.ppat.1008781 PMC7508367 32810179

[23] Hemmige V, Tanowitz H, Sethi A. *Trypanosoma cruzi* infection: A review with emphasis on cutaneous manifestations. Int J Dermatol. 2012;51(5):501–8. doi: 10.1111/j.1365-4632.2011.05380.x 22515575 PMC3552304

[24] Zulantay I, Apt W, Valencia C, Torres A, Saavedra M, Rodríguez J, et al. Detection of *Trypanosoma cruzi* in untreated chronic chagasic patients is improved by using three parasitological methods simultaneously. J Antimicrob Chemother. 2011;66(10):2224–6. doi: 10.1093/jac/dkr313 21798882

[25] Li Y, Shah-Simpson S, Okrah K, Belew AT, Choi J, Caradonna KL, et al. Transcriptome remodeling in *Trypanosoma cruzi* and human cells during intracellular infection. PLOS Pathog. 2016;12(4):e1005511. doi: 10.1371/journal.ppat.1005511 PMC4821583 27046031

